# Genetic Characteristics of Multiple Copies of Tn1546-Like Elements in ermB-Positive Methicillin-Resistant *Staphylococcus aureus* From Mainland China

**DOI:** 10.3389/fmicb.2022.814062

**Published:** 2022-02-28

**Authors:** Haiping Wang, Dandan Wu, Lingfang Di, Feiteng Zhu, Zhengan Wang, Lu Sun, Yiyi Chen, Shengnan Jiang, Hemu Zhuang, Mengzhen Chen, Shujuan Ji, Yan Chen

**Affiliations:** ^1^Department of Infectious Diseases, Sir Run Run Shaw Hospital, Zhejiang University School of Medicine, Hangzhou, China; ^2^Key Laboratory of Microbial Technology and Bioinformatics of Zhejiang Province, Hangzhou, China; ^3^Regional Medical Center for National Institute of Respiratory Diseases, Sir Run Run Shaw Hospital, Zhejiang University School of Medicine, Hangzhou, China; ^4^Department of Infectious Diseases, Second Affiliated Hospital, Zhejiang University School of Medicine, Hangzhou, China; ^5^Department of Clinical Laboratory, Tongxiang First people’s hospital, Tongxiang, China; ^6^Department of Hospital Epidemiology and Infection Control, Sir Run Run Shaw Hospital, Zhejiang University School of Medicine, Hangzhou, China

**Keywords:** methicillin-resistant *Staphylococcus aureus*, Tn1546-like elements, *ermB*-positive, multiple copies, clonal complex 5

## Abstract

**Objective:**

To determine the genetic structure of *ermB*-positive Tn1546-like mobile elements in methicillin-resistant *Staphylococcus aureus* (MRSA) from mainland China.

**Methods:**

A total of 271 erythromycin-resistant MRSA isolates were isolated from Sir Run Run Shaw Hospital (SRRSH) from 2013 to 2015. Whole-genome sequencing was performed for the *ermB*-positive strains, and the genetic environment of the *ermB* genes was analyzed. Southern hybridization analysis and transformation tests were performed to confirm the location of the *ermB* gene.

**Results:**

A total of 64 isolates (64/271, 23.6%) were *ermB*-positive strains, with 62 strains (62/64, 96.9%) belonging to the CC59 clone. The other two strains, SR130 and SR231, belonging to CC5-ST965, both harbored 14,567 bp *ermB*-positive Tn1546-like elements and displayed multidrug-resistant profiles. PFGE followed by Southern blot demonstrated that the *ermB* genes were located on the plasmids of both SR130 and SR231, while two copies of ermB were located on the chromosome of SR231. Further sequencing demonstrated that SR231 carried one Tn1546-*ermB* elements in the plasmid and two identical copies integrated on the chromosome, which had 99.99% identity to the element in the plasmid of SR130. The Tn1546-*ermB* elements were highly similar (100% coverage, >99.9% identity) to the element Tn6636 reported in a previous study from Taiwan. The plasmids (pSR130 and pSR231) harboring *ermB*-positive Tn1546-like elements were also identical to the mosaic plasmid pNTUH_5066148. However, conjugation of *ermB*-carrying plasmids of SR130 and SR231 were failed after triple repeats.

**Conclusion:**

Multiple copies of *ermB*-positive Tn1546-like mobile elements were found in CC5-ST965 MRSA from mainland China, showing the wide dissemination of these *Enterococcus faecium*-originated *ermB*-positive Tn1546-like elements. Molecular epidemiological study of Tn1546-like elements is essential to avoid the spreading of resistant determinants.

## Introduction

*Staphylococcus aureus* is an important Gram-positive pathogen that can cause a variety of infectious diseases and result in increased mortality (Su et al., 2013). The antibiotic resistance problem with *S. aureus* has attracted worldwide attention. In addition to methicillin-resistant *S. aureus* (MRSA), macrolide resistance is also a common and severe problem. A meta-analysis study in a healthy Chinese population found that 88% of MRSA isolates were resistant to erythromycin (Wu et al., 2019). Antimicrobial surveillance of bacterial isolates from patients hospitalized with community-acquired skin infections in Europe, Asia and Latin America showed that 56% of MRSA isolates were not susceptible to erythromycin (Sader et al., 2019). Thus, erythromycin resistance in MRSA is a problem that is worthy of attention.

As a kind of macrolide, erythromycin resistance in *S. aureus* is primarily due to ribosomal alteration of the 23S rRNA target site by methylases encoded by erm genes (Fessler et al., 2018). *ermB*, which was originally identified in *Enterococcus faecalis*, is now widespread in *S. aureus*. In 1999, it was first found on the transposon Tn551 in *S. aureus*, which consists of *ermB*, the resolvase gene *tnpR* and the transposase gene *tnpA* (Wu et al., 1999). However, recently, a novel structure of *ermB* carrying elements was found in *S. aureus* in Taiwan, China. It is a 14,566-bp element that has the structure of a Tn1546-like transposon with the *ermB* gene located within Tn551 (Wan et al., 2016), which was recently renamed Tn6636 (Wan et al., 2021). Previous reports have indicated that the horizontal transfer of Tn1546 plays an important role in the spread of VanA-type vancomycin-resistant *Enterococcus* (VRE) and vancomycin-resistant *S. aureus* (VRSA) (Perichon and Courvalin, 2009). Thus, the presence of Tn1546-like transposons in *S. aureus* calls for more attention.

In this study, we investigated the prevalence of the *ermB* gene among MRSA isolates isolated from 2013 to 2015 in Sir Run Run Shaw Hospital (SRRSH) and analyzed the structure of the mobile element harboring the *ermB* gene. We found two strains carrying *ermB*-positive Tn1546-like elements with high similarity to previous reports in Taiwan. To our knowledge, this is the first report of the *ermB*-positive Tn1546-like element found in MRSA isolates outside Taiwan Island.

## Materials and Methods

### Bacterial Isolates

A total of 271 erythromycin-resistant MRSA isolates were collected from SRRSH, Zhejiang University School of Medicine, Hangzhou, China, from January 1, 2013 to December 31, 2015. Molecular typing and susceptibility testing were performed as in previous reports (Chen et al., 2018).

### Antibiotic Resistance

Antimicrobial susceptibility testing was performed using Etest strips according to the instructions. Cefoxtin, vancomycin, teicoplanin, levofloxacin, ciprofloxacin, erythromycin, tetracycline, clindamycin, rifampicin, nitrofurantoin, fosfomycin, and linezolid were tested. The fosfomycin results were interpreted in accordance with the European Committee on Antimicrobial Susceptibility Testing (EUCAST), while the others were interpreted according to CLSI (2019).

### Genome Sequencing and Annotation

Genomic DNA was extracted via a QIAamp DNA minikit (Qiagen Valencia, CA, United States). The quality of DNA was determined by gel electrophoresis and a NanoDrop 2000 spectrophotometer. The genome was sequenced using a HiSeq X Ten platform (Illumina, San Diego, CA, United States) with 2 × 150 bp paired-end reads. The WGS data were used for spa typing and MLST, using SeqSphere + software with standard procedures. Nanopore sequencing using a MinION sequencer (Oxfors Nanopore Technologies, Oxford, United Kingdom) was performed for the genomic DNA of SR130 and SR231. Hybrid assembly was achieved with Unicycler using the Illumina reads and Nanopore reads (Wick et al., 2017). The genome sequences of the chromosome and plasmids were annotated using the RAST server (Aziz et al., 2008). Screening of the *ermB* genes was performed using ResFinder.^1^

### Genetic Structure of the ermB-Positive Mobile Elements

The map of the sequence comparison of the genetic structure of the *ermB*-positive mobile elements was generated via Easyfig 2.1 using the WGS data (Sullivan et al., 2011). The reference sequence of ST59-MSSA pNTUH_3874 (accession no. LC102479) was downloaded from NCBI.

### Pulsed-Field Gel Electrophoresis

Pulsed-field gel electrophoresis (PFGE)was performed according to the method described by Bannerman et al. (1995) with some modifications. Genomic DNA was prepared in agarose blocks and digested with *Sma*I. The DNA fragments were separated using a CHEF-Mapper XA PFGE System (Bio-Rad) for 22 h at 6 V cm^–1^ and 14°C, with a pulse angle of 120° and pulse times of 3–40 s. The PFGE banding patterns were analyzed visually according to the interpreting criteria described by Tenover et al. (1995).

### S1 Nuclease PFGE and Southern Blot Hybridization

To confirm where the *ermB* gene was located, S1 nuclease PFGE was performed (Barton et al., 1995). The DNA in gel plugs was digested at 37°C for 40 min with 20 units of S1 nuclease (TAKARA, Dalian, China). The plugs were applied to the wells of 1.0% agarose gels (Sangon Biotech, Shanghai, China) and run in a CHEF-Mapper XA PFGE system (Bio-Rad, United States). Electrophoresis was performed for 20 h at 14°C with pulse times ranging from 2.16 to 63.8 s at 6 V/cm in 0.5 × Tris-borate-EDTA (TBE) buffer. Each band was considered to be a unit length linear plasmid.

The DNA fragments of both PFGE and S1-PFGE were transferred horizontally to a nylon membrane (Millipore) and hybridized with a digoxigenin-labeled *ermB* probe using the DIG-High Prime DNA Labeling and Detection Starter KitII (Roche Diagnostics). The erythromycin-resistant MRSA strain SR144, with the *ermB* gene located on the chromosome, was used as the control strain.

### Plasmid Extraction and Electroporation

Plasmid DNA was extracted using a plasmid miniprep kit (Axygen, United States) and electroporated into *S. aureus* RN4220 as described previously (Schenk and Laddaga, 1992). Plasmid DNA (1–5 μg) was mixed with 100 μl of electrocompetent cells, transferred to a 1-mm electroporation cuvette and electroporated using a Bio-Rad gene pulser (Bio-Rad, United States) set at 2.5 kV, 200 Ω resistance and 25 μF capacitance. Transformants were expanded at 200 rpm for 3 h and selected on Tryptic Soy Agar plates containing erythromycin (4 μg/ml), and antimicrobial susceptibility testing was performed using the Etest strip method.

### Conjugative Assays

Filter-mating experiments were completed similar to a previous description (Forbes and Schaberg, 1983). Briefly, the rifampicin-resistant strain ATCC29213-rifR was used as the recipient in the filter-mating experiments, SR130 and SR231 were used as donors, and mating was carried out on a filter. After overnight mating, the filters were removed into EP tubes with 1 ml of TSB and vortexed vigorously for 1 min to release the cells. Twenty microliters of the cells were plated on Tryptic Soy Agar plates containing 5 μg/ml erythromycin for selection of the transconjugants. The transconjugants were also checked by PCR and sequencing for the *pta* housekeeping gene.

### Nucleotide Sequence Accession Number

The complete sequences of the plasmids pSR130 and pSR231 were deposited at NCBI under the following accession numbers: CP047925 and CP047923, respectively. The chromosome sequence of SR130 and SR231 was deposited at NCBI under accession numbers CP047924 and CP047922, respectively.

## Results

### Prevalence of ermB, the Clinical Characterization and Molecular Epidemiology of SR130 and SR231

Among the 271 erythromycin-resistant MRSA isolates, 64 isolates (64/271, 23.6%) were *ermB*-positive strains. Most (62/64, 96.9%) of the *ermB*-positive MRSA belonged to the CC59 clone, except for SR130 and SR231 (2/64, 3.1%). The SR130 and SR231 strains were both collected in 2013. SR130 was derived from the sputum of a patient with a respiratory infection, and SR231 was obtained from the secretions of a patient with SSTI. SR130 and SR231 had the same antimicrobial susceptibility pattern and were resistant to cefoxitin, levofloxacin, ciprofloxacin, erythromycin, clindamycin, and gentamicin but susceptible to vancomycin, teicoplanin, tetracyclin, rifampicin, nitrofurantoin, fosfomycin and linezolid (Table 1).

**TABLE 1:** Antimicrobial susceptibility patterns of SR130, SR 231, RN4220 and the transformants of pSR130-RN4220 and pSR231-RN4220.

Antimicrobial agent	MIC (mg/L)				
	SR130	SR231	pSR130-RN4220	pSR231-RN4220	RN4220
Cefoxtin	32	64	2	1.5	1.5
Vancomycin	1	1	1.5	2	2
Teicoplanin	0.5	1	0.5	0.38	0.38
Levofloxacin	8	8	0.5	0.25	0.25
Ciprofloxacin	32	64	0.38	0.38	0.38
Erythromycin	>256	>256	>256	>256	0.5
Tetracycline	0.25	0.25	0.38	0.38	0.25
Clindamycin	>256	>256	>256	>256	0.125
Rifampicin	0.006	0.006	0.008	0.008	0.008
Nitrofurantoin	8	8	4	4	4
Fosfomycin	0.5	0.25	1.5	0.5	0.5
Linezolid	2	1	2	2	2
Gentamicin	32	32	24	24	1

**FIGURE 1:**
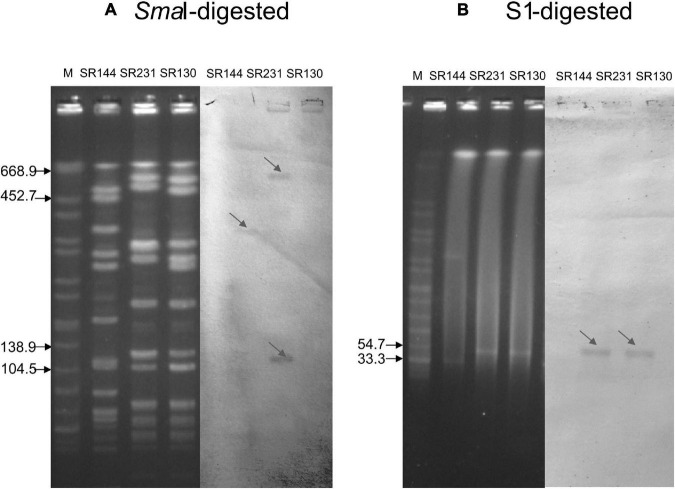
Southern blot analysis of the *ermB* gene. **(A)** PFGE for *Sma*I-digested DNA (left four panels) and Southern blot hybridization for the *ermB* gene (right three panels). **(B)** PFGE for S1-digested plasmid DNA (left four panels) and Southern blot hybridization for the *ermB* gene (right three panels). The bands with red arrows showed positive signals by Southern blot hybridization with the *ermB* probe. SR144 with *ermB* carried on the chromosome but not in the plasmid was used as a negative control in S1 and a positive control on the chromosome. Salmonella serotype Braenderup strain H9812 was used as a molecular marker. The results showed that the *ermB* gene is located in the 33.3–54.7 kb plasmid in SR130 and SR 231, and SR 231 also carried two copies of *ermB* genes on the chromosome.

Both the SR130 and SR231 strains belonged to the CC5-ST965-t602 clone. The PFGE pattern showed five band differences between SR130 and SR231, which were considered to be possibly related to each other (Figure 1A).

### Location of the ermB Genes, Transformation Tests and Conjugation Tests

S1-PFGE followed by Southern blot demonstrated that the *ermB* genes were located on the plasmids of both SR130 and SR231, with an approximately equal size around 33.3–54.7 kb. However, *Sma*I-PFGE followed by Southern blot demonstrated that two copies of *ermB* were located on the chromosome of SR231. This means that SR231 harbors three copies of *ermB* genes, two copies located on the chromosome and one copy located on the plasmid (Figures 1A,B).

The *ermB*-carrying plasmids were successfully transferred from SR130 and SR231 to laboratory strain ST5-MSSA RN4220 by electroporation. PCR results confirmed that *ermB* genes were detected in the transformants. Compared with RN4220, the transformants exhibited increased MICs for erythromycin (>256 μg/mL), clindamycin (>256 μg/mL), and gentamicin (24 μg/mL) (Table 1). However, conjugation of *ermB*-carrying plasmids to the rifampicin-resistant strain ATCC29213-rifR failed after triple repeats.

### Genetic Structure of the ermB-Positive Mobile Elements

**FIGURE 2:**
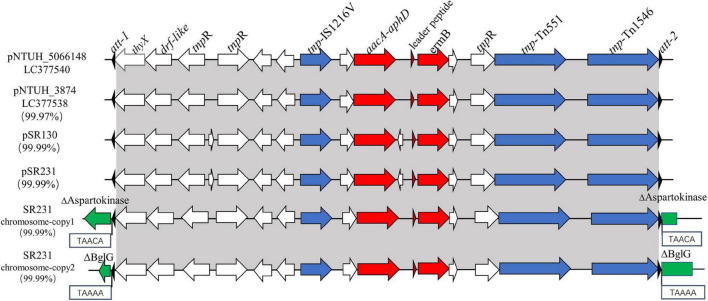
Genetic structures of *ermB*-positive Tn1546-like elements in pSR130, pSR231 and in two different positions of the SR231 chromosome and structural comparison with pNTUH_3874 and pNTUH_5066148. *thyX*, thymidylate synthase; *drf-like*, dihydrofolate reductase; *ermB*, conferring resistance to erythromycin; *aacA-*aphD, conferring resistance to kanamycin/gentamicin.

In all 62 erythromycin-resistant CC59-MRSA isolates, the *ermB* gene was located on transposon Tn551 with an interrupted *sasK* gene in the chromosome, as previously reported (Wan et al., 2016). However, all of the *ermB* genes in SR130 and SR231, both in the plasmids or in the chromosome, were located in Tn1546-like elements (Figure 2).

The 14,567 bp *ermB*-positive Tn1546-like mobile elements in SR231, one copy in the plasmid and two copies in the chromosome, were 99.99% identical to the sequences of elements in SR130. In addition to the *ermB* gene, it contains another antimicrobial resistance gene *aacA-aphD* (gentamicin/kanamycin resistance), one insertion sequence IS1216 V, two transposase genes (*tnp*) (Tn1546 and Tn551), three resolvase genes (*tnpR*), one leader peptide of the *ermB* gene, one thymidylate synthase gene (*thyX*), and one dihydrofolate reductase gene (*drf-like*) (Figure 2). It was highly similar to the *ermB*-positive mobile elements found in the plasmids reported on Taiwan Island (Wan et al., 2016), including pNTUH_3874 (100% coverage, 99.91% identity, accession no. LC102479) (Figure 2), pNTUH_5066148 (100% coverage, 99.99% identity, accession no. LC377540), pNTUH_9448 (100% coverage, 99.98% identity, accession no. LC377536) and pNTUH_1027 (100% coverage, 99.99% identity, accession no. LC377537).

### Two Copies of ermB-Positive Tn1546-Like Elements in the Chromosome of SR231

Previous studies have shown that the Tn551 element carrying the *ermB* gene is usually inserted into the *sasK* gene. For SR231, the intact *sasK* gene was detected on its chromosome, and two copies of *ermB*-positive Tn1546-like elements were inserted into a 5-bp suspected target sequence (TAAAA and TAACA) in genes coding for BglG and aspartokinase, which are related to sugar metabolism and amino acid metabolism, respectively (Figure 2). Both of the elements were flanked by two 38-bp transposase distal terminal inverted repeats (att-1 sequence 5′-GGGGTAGCGTCAGGAAAATGCGGATTTACAACGTTAAG-3′, att-2 sequence 5′-CTTAGCGTTGTAAATCCGCATTTTCCT GACGGTACCCC-3′) at the ends. Although they had high homology, the genes for BglG and aspartokinase in SR130 were intact.

### Mosaic Plasmids Harboring the ermB-Positive Tn1546-Like Element

Each of the SR130 and SR231 strains carried one plasmid, designated pSR130 and pSR231, respectively. Comparing the sequences of these two plasmids showed that they were identical (100% coverage and 100% identity) and 39227 bp in size. The plasmid was comprised of 54 putative open reading frames (ORFs), and the structures of mosaic plasmids pSR130 and pSR231 were almost the same as that of the plasmid pNTUH_5066148 (100% coverage, 99.97% identity, accession no. LC377540) from a ST965-MRSA in Taiwan Island.

The backbone of pSR130 and pSR231 was 24,660 bp in length. It was of high similarity (100% coverage, >99.8% identity) to several plasmids isolated in the USA (CA-347 plasmid, pER05295.3A.1, pER05215.3A.1, pER07317.3A.1), Korea (pFORC_090.1), Japan (pN315), China (pSR03) and Taiwan Island (pNTUH_5066148). The repA genes in pSR130 and pSR231 were also of high similarity (100% coverage, ≥99.5% identity) to those in the plasmids above. As mosaic plasmids, *ermB*-positive Tn1546-like elements were inserted into the backbone of pSR130 and pSR231 with the disruption of one of the three *rep* genes, shortening the amino acid length from 286 to 270 (Supplementary Figure 1).

## Discussion

The *ermB* gene is one of the most prevalent determinants conferring resistance to macrolides in *S. aureus*. Although most of the *ermB* genes in *S. aureus* are located on transposon Tn551, a novel *ermB*-carrying element has been described in Taiwan (Wan et al., 2016). In this study, we reported two ST965 erythromycin-resistant MRSA strains harboring *ermB*-positive Tn1546-like elements in plasmids and in the chromosome, which is the first report about Tn1546-*ermB* elements in mainland China.

Tn1546, a mobile element first found in enterococci, was demonstrated to be responsible for the transfer of the vanA operon from VRE to *S. aureus* and this led to the emergence of VRSA (Gardete and Tomasz, 2014). The *ermB*-positive Tn1546-like mobile elements in our research were located in two identical plasmids, pSR130 and pSR231, which are highly similar to pNTUH_5066148, a plasmid harboring *ermB*-positive Tn1546-like elements from Taiwan Island. pNTUH_5066148 has been demonstrated to have the ability to be transferred by conjugation (Wan et al., 2021). However, the conjugation test failed in our research, which may be explained by the relatively low conjugation frequency. Moreover, the backbones of the plasmids above are highly similar to many plasmids, including pCA-347 and pN315, which are widespread throughout the world and may facilitate the horizontal transmission of Tn1546-like elements.

In the present study, both Tn1546-*ermB*-positive isolates SR130 and SR231 were ST965-MRSA strains. ST965 is a single-locus variant of ST5 that belongs to clonal complex 5 (CC5). CC5-MRSA is the most common and widespread MRSA lineage and has increasingly been isolated from China and abroad (Chen et al., 2018; Perez-Montarelo et al., 2018; Wu et al., 2018; Vazquez-Rosas et al., 2021). Meanwhile, most VRSA isolates with typing data are categorized into the CC5 lineage, which independently acquires vanA-positive Tn1546 from enterococci (Limbago et al., 2014; Challagundla et al., 2018). It seems that the CC5 lineage is more suitable for capturing the Tn1546 element; however, the underlying mechanisms have not been fully understood to date (Kos et al., 2012; Cong et al., 2020).

Due to the development of sequencing technology, multiple copies of resistance genes located in a single strain have been increasingly reported recently. Some studies have demonstrated that harboring multiple copies of a resistance gene in a single strain can increase the expression of the resistance gene and exacerbate antimicrobial resistance (Gallagher et al., 2017). In our research, PFGE followed by Southern blot identified that multiple copies of *ermB* genes existed in SR231, and further sequencing confirmed that three copies of *ermB*-positive Tn1546-like elements located in SR231, with double copies inserted in two different positions in the chromosome and one copy in the plasmid. However, no increase in erythromycin and clindamycin resistance was seen between SR231 and other strains harboring single copies of *ermB*, including SR130, pSR130-RN4220 and pSR130-RN4220. The reason might be that *ermB* confers high-level resistance to macrolide, lincosamide and streptogramin B, even with only one copy, so no noticeable link between the copy number and antimicrobial resistance was seen in our results. On the other hand, as multiple copies of Tn1546-like elements were inserted in CC5-ST965-MRSA strain SR231, it seemed the hotspots where Tn1546-like elements can insert were varied, which may facilitate the transfer of this element between chromosomes and plasmids, especially in the CC5 clone, which has drawn much attention. The transmission of Tn1546-like elements or Tn1546-harboring plasmids among *S. aureus* isolates may increase the possibility of vanA operon integration into *S. aureus*, which may subsequently lead to the emergence and spreading of VRSA (Perichon and Courvalin, 2009), and thus monitoring the occurrence of Tn1546-like elements in *S. aureus* is crucial. Beside the 271 erythromycin-resistant MRSA isolates from SRRSH, we searched Tn1546 sequences in 471 sequenced MRSA isolates from our national prevalence study, all the Tn1546 elements were found located on the plasmids, except SR231 (Supplementary Table 2). Additionally, we searched Tn1546 sequences in NCBI database by BLAST, and we found three ST398 *S. aureus* isolates from Canada and one ST1 isolate from China had Tn1546 in their chromosomes, indicating that these elements rarely integrated into chromosomes so far.

## Conclusion

Multiple copies of *ermB*-positive Tn1546-like mobile elements were detected in CC5-ST965 MRSA in mainland China, showing the wide dissemination of these *Enterococcus faecium*-originated ermB-positive Tn1546-like elements. The transmission of Tn1546-like elements in *S. aureus* may increase the possibility of the vanA operon integrating into *S. aureus*. Therefore, special attention should be given to monitoring the prevalence of Tn1546-like elements in *S. aureus*.

## Data Availability Statement

The names of the repository/repositories and accession number(s) can be found below: https://www.ncbi.nlm.nih.gov/, CP047924–CP047925 and https://www.ncbi.nlm.nih.gov/, CP047922–CP047923.

## Ethics Statement

This study was approved by the local Ethics Committee in Sir Run Run Shaw Hospital with a waiver of informed consent (Approval No.20150115-1). Written informed consent for participation was not required for this study in accordance with the national legislation and the institutional requirements.

## Author Contributions

YC, SJJ, and HW designed the study. HW, DW, and LD drafted the manuscript. FZ, HZ, ZW, SNJ and MC did the experiments. LS, YYC, SJJ, and YC revised the manuscript. All authors have approved the final manuscript.

## Conflict of Interest

The authors declare that the research was conducted in the absence of any commercial or financial relationships that could be construed as a potential conflict of interest.

## Publisher’s Note

All claims expressed in this article are solely those of the authors and do not necessarily represent those of their affiliated organizations, or those of the publisher, the editors and the reviewers. Any product that may be evaluated in this article, or claim that may be made by its manufacturer, is not guaranteed or endorsed by the publisher.

## Footnotes

1
http://www.genomicepidemiology.org
