# Supplementary material for: Genetic Characteristics of Multiple Copies of Tn1546-Like Elements in ermB-Positive Methicillin-Resistant Staphylococcus aureus From Mainland China
Source: Front Microbiol. 2022 Feb 28;13:814062. doi: 10.3389/fmicb.2022.814062 (PMC8919048; doi:10.3389/fmicb.2022.814062)
Supplement: Supplementary file 1 [file Data_Sheet_1.PDF]

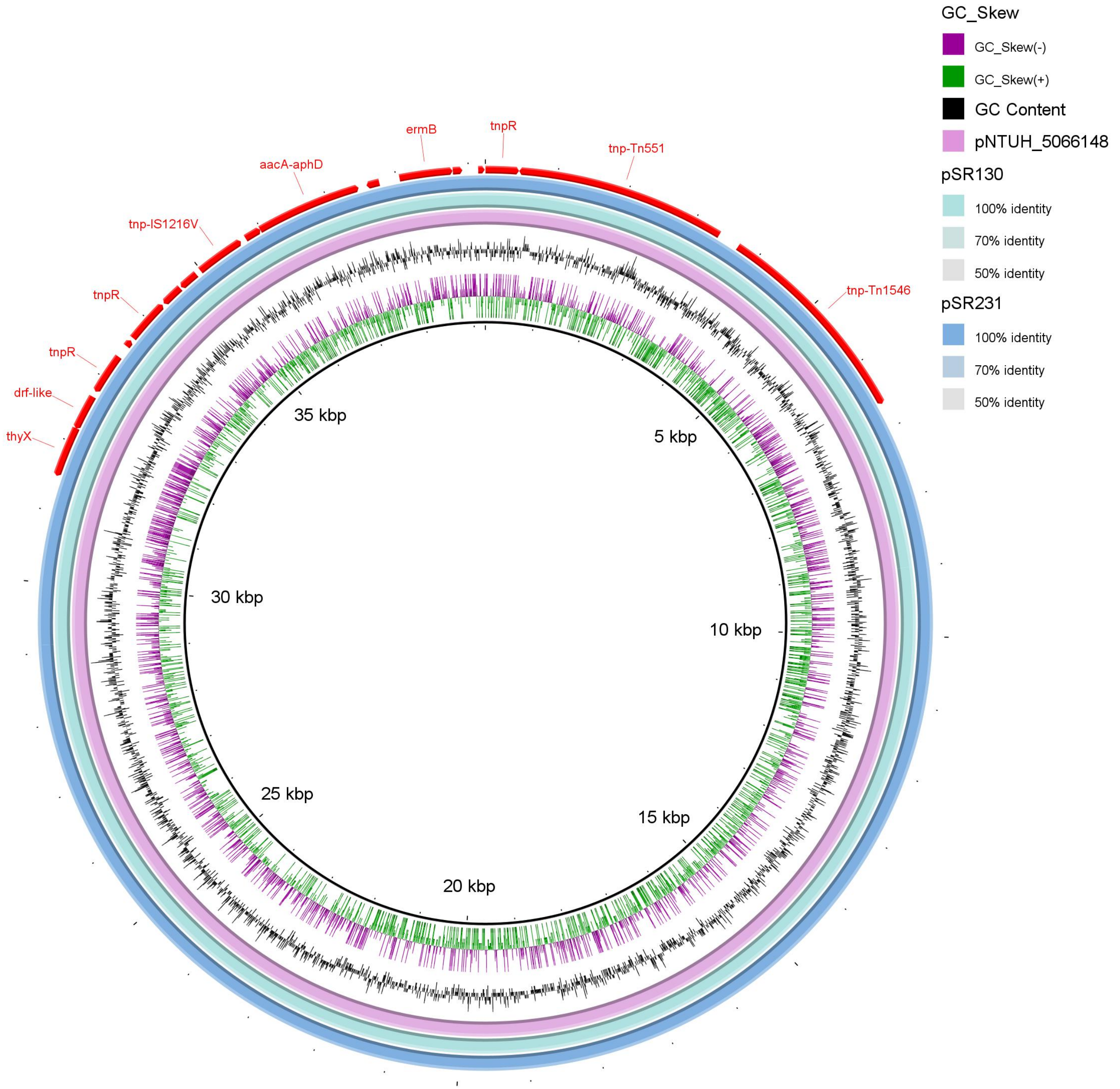

Figure S1. Circular map of plasmids pSR130 and pSR231 compared with pNTUH_5066148 (accession no. LC377540). The red curve indicates the position and the structure of the Tn1546-like elements.
