# Supplementary material for: Genetic Characteristics of Multiple Copies of Tn1546-Like Elements in ermB-Positive Methicillin-Resistant Staphylococcus aureus From Mainland China
Source: Front Microbiol. 2022 Feb 28;13:814062. doi: 10.3389/fmicb.2022.814062 (PMC8919048; doi:10.3389/fmicb.2022.814062)
Supplement: Supplementary file 2 [file Data_Sheet_2.docx]

Figure S2. The complete chromosomes sequence of SR231 and SR130 were compared using Mauve (version 2.4.0) with SR231 as the reference.


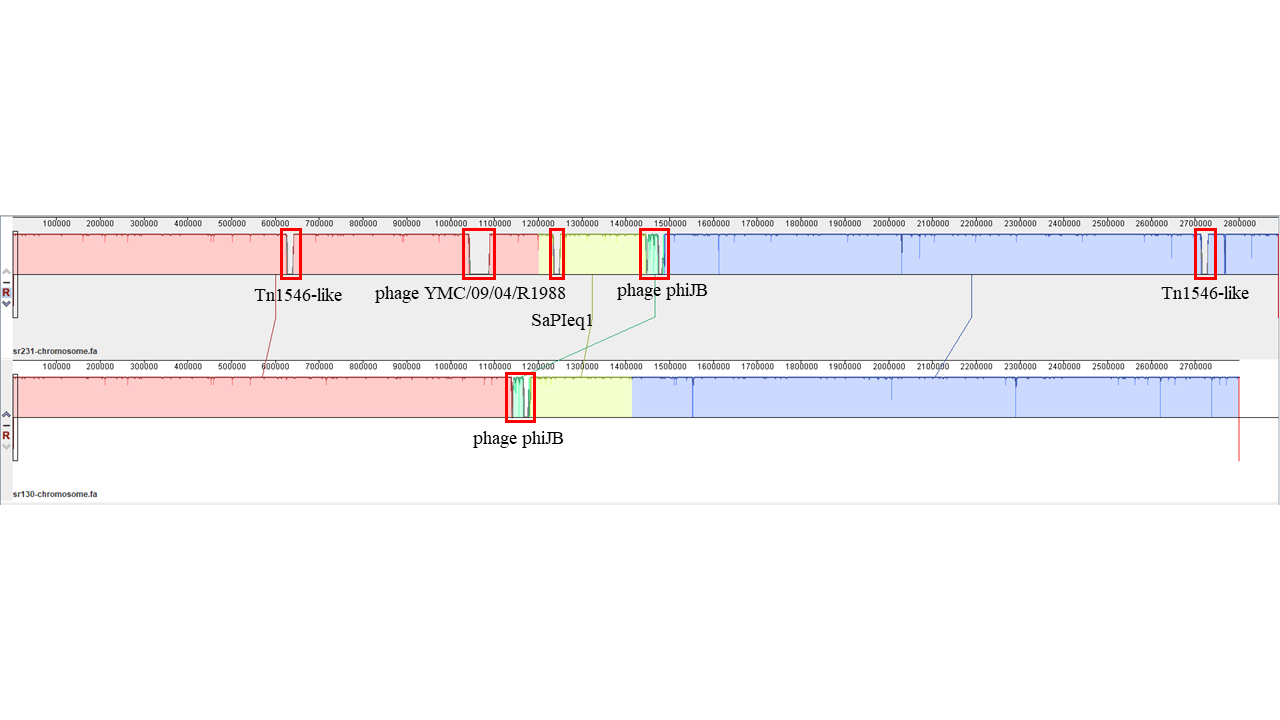


No rearrangement was detected and the difference between these two isolates were mainly on mobile genetic elements. The phage phiJB was contained by two strains but at different position and different inner sequence. SR231 acquired other three mobile elements, including phage YMC/09/04/R1998, virulence island, and two copies of Tn1546-lke.
