# Supplementary material for: Genetic Characteristics of Multiple Copies of Tn1546-Like Elements in ermB-Positive Methicillin-Resistant Staphylococcus aureus From Mainland China
Source: Front Microbiol. 2022 Feb 28;13:814062. doi: 10.3389/fmicb.2022.814062 (PMC8919048; doi:10.3389/fmicb.2022.814062)
Supplement: Supplementary file 3 [file Data_Sheet_3.docx]

**Tree scale: 0.00001**

Figure S3. The phylogenetic tree of ST965 MRSA isolates carried Tn1546 from mainland China

**location of Tn1546**

plasmid

chromosome and plasmid negative

N26CSA20 N28CSA24 SR130 SR231 N28HSA16 N28HSA03 N28HSA19 N28CSA30 N28HSA11 SR262 SR251 SR253
