# Supplementary material for: Genetic Characteristics of Multiple Copies of Tn1546-Like Elements in ermB-Positive Methicillin-Resistant Staphylococcus aureus From Mainland China
Source: Front Microbiol. 2022 Feb 28;13:814062. doi: 10.3389/fmicb.2022.814062 (PMC8919048; doi:10.3389/fmicb.2022.814062)
Supplement: Supplementary file 4 [file Table_1.DOCX]

| **Table S1. Clindamycin resistance phenotype and resistance genes of MRSA isolates** | | | | | | | | | | | | |
| --- | --- | --- | --- | --- | --- | --- | --- | --- | --- | --- | --- | --- |
|  |  |  | Resistance genes | | | | | | | Phenotype | | |
| ST | NO | SCC*mec* | *ermA* | *ermB* | *ermC* | *ermA+ermC* | *ermA+lnuA* | *ermB+lnuA* | *ermC+lnuA* | iMLS | cMLS | CS |
| ST59 | 68 | Ⅳ(51),Ⅴ(17) | - | 54(79.4) | 6(8.8) | - | - | 1(1.5) | - | 1(1.5) | 60(88.2) | 7(10.3) |
| ST5 | 150 | Ⅱ(148),Ⅳ(1),NT(1) | 51(34.0) | - | 1(0.7) | - | 98(65.3) | - | - | 123(82) | 27(18) | 0(0) |
| ST239 | 19 | Ⅲ(16),NT(3) | 10(52.6) | - | 2(10.5) | 7(36.8) | - | - | - | 2(10.5) | 15(79) | 2^a^(10.5) |
| ST630 | 10 | Ⅴ(10) | - | - | 8(80.0) | - | - | - | - | 8(80.0) | - | 2(20.0) |
| ST88 | 8 | Ⅳ(4),NT(4) | - | - | 4(50.0) | - | - | - | 3(37.5) | 7(87.5) | - | 1(12.5) |
| ST965 | 5 | Ⅳ(5) | - | 2(40.0) | 3(60.0) | - | - | - | - | 3(60.0) | 2(40.0) | - |
| ST338 | 4 | Ⅴ(4) | - | 4(100.0) | - | - | - | - | - | - | 4(100.0) | - |
| ST25 | 3 | NT(3) | - | - | 1(33.3) | - | - | - | - | - | 1(33.3) | 2(66.7) |
| ST398 | 3 | Ⅴ(3) | - | - | 2(66.7) | - | - | - | - | 2(66.7) | - | 1(33.3) |
| ST1 | 3 | Ⅳ(3) | - | - | 3(100.0) | - | - | - | - | 3(100.0) | - | - |
| ST22 | 2 | Ⅴ(2) | - | - | 2(100.0) | - | - | - | - | 2(100.0) | - | - |
| ST1611 | 2 | Ⅳ(2) | - | - | 1(50.0) | - | - | - | - | 1(50.0) | - | 1(50.0) |
| ST6174 | 2 | Ⅳ(2) | - | - | - | - | - | - | - | - | - | 2(100.0) |
| Other^b^ | 13 | Ⅱ(4),Ⅳ(6),Ⅴ(2),NT(1) | 3(23.0) | 3(23.0) | 2(15.4) | - | 1(7.7) | - | - | 3(23.0) | 6(46.2) | 4(30.8) |
| a: Two ST239 isolates were *ermA* positive but susceptible to clindamycin.  b: one isolate for each ST，including ST20, ST772, ST950, ST3194, ST3195, ST4513, ST4988, ST5530, ST6173, ST6175, ST6190, ST6191, and ST6192. | | | | | | | | | | | | |

**Table S2.** **Distribution of MLST and plasmid-backbone in Tn1546-like**

**carrying MRSA from China**

| **strain** | **ST** | **plasmid-backbone** | **accession NO** |
| --- | --- | --- | --- |
| N24HSA33 | 1 | pSaa6159 | CP002115 |
| N24HSA04 | 1 |  |  |
| N27HSA12 | 7 |  |  |
| N12CSA36 | 88 |  |  |
| N10CSA09 | 88 |  |  |
| N16HSA02 | 188 |  |  |
| N27CSA05 | 5540 |  |  |
|  |  |  |  |
| N26HSA44 | 5 | pWBG744 | GQ900398 |
| N20CSA14 | 5 |  |  |
|  |  |  |  |
| N28CSA24 | 965 | pCA-347/pN315 | CP006045/AP003139 |
| N26CSA20 | 965 |  |  |
| N28HSA19 | 965 |  |  |
| N28HSA16 | 965 |  |  |
| N28CSA30 | 965 |  |  |
| N28HSA11 | 965 |  |  |
| N28HSA03 | 965 |  |  |
| N28CSA22 | 5531 |  |  |
| SR231 | 965 |  |  |
| SR130 | 965 |  |  |

**Table S3.** **Distribution of MLST in Tn1546-like carrying *S.aureus* from NCBI database**

| **genome sequence** | **strain** | **ST** | **country** | **PMID** | **accession NO** |
| --- | --- | --- | --- | --- | --- |
| chromosome | GDY8P96A | 1 | China.Guangzhou | Unpublished | CP065515 |
| chromosome | GD1706 | 398 | Canada | Unpublished | CP040232 |
| chromosome | GD1696 | 398 | Canada | 31296690 | CP040233 |
| chromosome | GD1677 | 398 | Canada | Unpublished | CP019595 |
| plasmid | NTUH_9448 | 5 | China.Taiwan | 33840606 | LC377536 |
| plasmid | NTUH_6457 | 188 | China.Taiwan | 33840606 | LC377539 |
| plasmid | NTUH_1027 | 7 | China.Taiwan | 33840606 | LC377537 |
| plasmid | NTUH_5066148 | 965 | China.Taiwan | 33840606 | LC377540 |
| plasmid | NTUH_3874 | 59 | China.Taiwan | 33840606 | LC570860 |
| plasmid | FORC59 | 188 | Korea | Unpublished | CP020355 |
| plasmid | FORC_039 | 188 | Korea | Unpublished | CP015818 |
